# Association between Gene Expression Profiles and Clinical Outcome of Pemetrexed-Based Treatment in Patients with Advanced Non-Squamous Non-Small Cell Lung Cancer: Exploratory Results from a Phase II Study

**DOI:** 10.1371/journal.pone.0107455

**Published:** 2014-09-24

**Authors:** Dean A. Fennell, Scott P. Myrand, Tuan S. Nguyen, David Ferry, Keith M. Kerr, Perry Maxwell, Stephen D. Moore, Carla Visseren-Grul, Mayukh Das, Marianne C. Nicolson

**Affiliations:** 1 Leicester University Hospital, Leicester, United Kingdom; 2 Eli Lilly and Company, Indianapolis, IN, United States of America; 3 New Cross Hospital, Wolverhampton, United Kingdom; 4 Aberdeen Royal Infirmary, University of Aberdeen, Aberdeen, United Kingdom; 5 Queens University, Belfast, United Kingdom; 6 Almac Diagnostics, Craigavon, United Kingdom; 7 Eli Lilly and Company, Houten, Netherlands; 8 Eli Lilly and Company, Basingstoke, United Kingdom; West German Cancer Center, Germany

## Abstract

**Introduction:**

We report exploratory gene-expression profiling data from a single-arm Phase-II-study in patients with non-squamous (ns)NSCLC treated with pemetrexed and cisplatin. Previously disclosed results indicated a significant association of low thymidylate-synthase (TS)-expression with longer progression-free and overall survival (PFS/OS).

**Methods:**

Treatment-naïve nsNSCLC patients (IIIB/IV) received 4 cycles of pemetrexed/cisplatin; non-progressing patients continued on pemetrexed-maintenance. Diagnostic tissue-samples were used to assess TS-expression by immunohistochemistry (IHC) and mRNA-expression array-profiling (1,030 lung cancer-specific genes). Cox proportional-hazard models were applied to explore the association between each gene and PFS/OS. Genes significantly correlated with PFS/OS were further correlated with TS-protein expression (Spearman-rank). Unsupervised clustering was applied to all evaluable samples (n = 51) for all 1,030 genes and an overlapping 870-gene subset associated with adenocarcinoma (ADC, n = 47).

**Results:**

51/70 tissue-samples (72.9%) were evaluable; 9 of 1,030 genes were significantly associated with PFS/OS (unadjusted p<0.01, genes: Chromosome 16 open reading frame 89, napsin A, surfactant protein B, aquaporin 4, TRAF2- and Nck-interacting kinase, Lysophosphatidylcholine acyltransferase 1, Interleukin 1 receptor type II, NK2 homeobox 1, ABO glycosyl-transferase); expression for all except IL1R2 correlated negatively with nuclear TS-expression (statistically significant for 5/8 genes, unadjusted p<0.01). Cluster-analysis based on 1,030 genes revealed no clear trend regarding PFS/OS; the ADC-based cluster analysis identified 3 groups (n = 21/11/15) with median (95%CI) PFS of 8.1(6.9,NE)/2.4(1.2,NE)/4.4(1.2,NE) months and OS of 20.3(17.5,NE)/4.3(1.4,NE)/8.3(3.9,NE) months, respectively.

**Conclusions:**

These exploratory gene-expression profiling results describe genes potentially linked to low TS-expression. Nine genes were significantly associated with PFS/OS but could not be differentiated as prognostic or predictive as this was a single-arm study. Although these hypotheses-generating results are interesting, they provide no evidence to change the current histology-based treatment approach with pemetrexed.

## Introduction

A major goal of current cancer research is to classify tumors by intrinsic characteristics and by expression of biomarkers that may predict response to chemotherapy. For patients with advanced NSCLC (if unselected for EGFR and ALK mutation status), platinum-based doublet chemotherapy remains the first-line standard of care. Clinical outcome of systemic therapy can be optimized by tailoring based on tumor histology or specific driver genetic alterations (e.g., EGFR mutation, ALK fusion/mutation). For patients with NSCLC of non-squamous (ns) histology, pemetrexed in combination with cisplatin has been associated with improved median survival when compared to gemcitabine plus cisplatin [1]. In patients with disease control and performance status 0–1 after 4 cycles of first-line pemetrexed/cisplatin treatment, maintenance treatment with pemetrexed may additionally improve survival [2].

The principal target of pemetrexed, a multi-targeted antifolate that gains entry to the cell via the reduced folate carrier, is thymidylate synthase (TS) [3]. TS is responsible for the conversion of deoxyuridine monophosphate to deoxythymidine monophosphate required for DNA replication and its inhibition eventually results in cell death. Earlier preclinical data suggest a potential association between overexpression of TS and reduced sensitivity to pemetrexed in antifolate-resistant cell lines [4], [5]. TS mRNA and protein expression is higher in squamous cell than in adenocarcinoma histopathological specimens [6].

We have recently published the primary results of a Phase II, single arm study of first-line pemetrexed/cisplatin in 70 patients with advanced nsNSCLC, followed by maintenance pemetrexed in 43 non-progressing patients, providing hypothesis-generating evidence that low TS expression both at the mRNA and protein level was associated with longer progression-free survival (PFS) and overall survival (OS) [7]. This evidence has recently been supported by additional pre-clinical and retrospective data [8]–[10]. In our study, median PFS was 5.5 months, median OS was 9.6 months from the start of induction treatment. The association between low TS expression and longer PFS/OS was most pronounced for TS protein expression in the nucleus, assessed by immunohistochemistry (IHC). Trends were similar for cytoplasm H-scores, quantitative reverse-transcriptase polymerase chain reaction (qPCR) and other clinical endpoints (response rate, and disease control rate).

Here, we report exploratory gene expression profiling data from this study, using a broad set of NSCLC-associated genes, providing the first gene expression profiling data of patients with advanced nsNSCLC treated with pemetrexed and cisplatin. This research was conducted to better understand the molecular characteristics of advanced nsNSCLC tumors with low TS expression, and to describe genes potentially linked to low TS expression. We aimed to generate evidence at multiple levels – protein, mRNA, gene expression profiles, and individual genes – for correlations with clinical outcome.

## Patients and Methods

### Ethics Statement

Written informed consent was obtained from all patients. The study was approved by the South Central Research Ethics Committee, Bristol, UK, and the Clinical Research Ethics Committee, Lancaster Hall, Cork, Ireland, and was performed in compliance with Good Clinical Practice.

### Study Design and Patients

H3E-BP-JMIK (NCT00887549) was an exploratory, single-arm, multicenter Phase II study, designed to assess prospectively the association between TS expression and clinical outcome. Treatment-naïve patients with nsNSCLC received up to 4 cycles (n = 70) of first-line pemetrexed/cisplatin, and non-progressing patients continued on pemetrexed maintenance (n = 43) treatment [7]; Figure S1 presents the study design. The primary results were recently published [7]. Secondary objectives involved exploration of gene expression data to correlate with survival (PFS, OS) – which are reported here.

### Tissue samples and assessment of gene expression

Collection of a diagnostic histological tumor sample was mandatory for each patient. All histopathological diagnoses were centrally reviewed by an expert pathologist, blinded to additional diagnostic information. Formalin-fixed, paraffin-embedded diagnostic tumor tissue (FFPE) samples were sent to a central laboratory, Almac Diagnostics, Craigavon, UK, who coordinated all laboratory work conducted under Almac standard operating procedures. As previously reported, the FFPE samples were used to evaluate TS expression on the protein and mRNA level [7].

Total RNA was extracted from each FFPE tissue sample of adequate quality, using 3–5 slides per RNA sample. Total RNA was processed for gene expression profiling analysis according to NuGEN and Affymetrix protocols for amplification, fragmentation, labeling, hybridization, washing, staining, and scanning.

#### Lung Cancer-DSA Research Tool

The Lung Cancer-DSA disease-specific array (LC-DSA) was used for mRNA profiling. This high density microarray platform (∼60,000 transcripts) was developed by Almac Diagnostics, Craigavon, UK using the standard Affymetrix Genechip platform methodology, providing a comprehensive and biologically relevant platform for the transcriptome of NSCLC [11].

#### mRNA expression assay

Resultant fragmented and labeled cDNA product was added to the hybridization cocktail in accordance with NuGEN guidelines for Affymetrix array hybridizations. The cocktail was hybridized for 16–18 hours to the LC-DSA using the GeneChip Hybridization Oven 450 (Affymetrix). Following hybridization the arrays were washed and stained using the GeneChip Fluidics Station 450 operated under the appropriate script (Affymetrix). The arrays were then scanned using the GeneChip Scanner 3000 (Affymetrix) to create the raw expression data.

#### Quality control

Only RNA samples and microarray profiling data which passed several quality assessments were used for further analysis (for details see Figure S2).

#### Normalization of raw data

The raw probe-level intensity measurement microarray data were normalized using the Robust Multichip Average (RMA) approach [12], [13].

#### Identification of most variable genes

A combined background and variance filter was applied to the data matrix to identify the most variable genes using an Almac-in-house developed feature selection program (details provided in File S1). In brief, to select the most variable genes, i.e. genes reflective of the main biology in the dataset, firstly a background filter was applied to remove genes with expression values too low to be distinguished from the background noise. In our approach, a high threshold was used to remove a large number of probe sets and ensure the selected probe sets are highly expressed (threshold: α = 0.001). Secondly, an intensity dependent variance filter was applied to the data matrix to only retain probe sets with high variance across all samples (threshold: α = 1.28×10^−12^). Feature selection resulted in 1462 most variable probe sets which were then summarized to a list of 1,030 genes. For gene transcripts expressed on different probe sets expression was summarized by using the median. All subsequent analyses were based on the summarized list of 1,030 genes.

### Statistical methods

#### Statistical software

mRNA profiles and their association with clinical outcomes were analyzed using the R open-source software version 2.15.1 (http://www.R-project.org).

#### Association of individual gene expressions with PFS, OS, and TS expression

A Cox proportional hazards regression model was applied to each of the 1,030 selected array genes (one gene at a time), to explore if their RMA expression was associated with PFS and OS. Two different models were used for each gene and endpoint. The first model included gene expression as a continuous variable (hazard ratio [HR] per 1-unit increase of RMA gene expression). The second model included gene expression as binary variable, after dividing RMA gene expression into high and low expression groups using the median of the gene expression distribution as cut-point. No additional covariates were used. In all models, a HR <1 indicated that higher gene expression was associated with longer PFS or OS. Due to the small sample size, multi-marker approaches were not considered for correlative analyses.

For unadjusted p-values, a significance level of 0.01 was used. To account for the multiple comparisons made across the 1,030 different genes for each endpoint and model, the False Discovery Rate (FDR) approach as described by Benjamini and Hochberg was additionally applied [14]. No further multiplicity adjustments were performed.

Genes significantly correlated with PFS and OS based on 2 different Cox-regression analyses (gene expression included as continuous or as binary variable using the median as cutpoint) were further correlated with nuclear TS-protein expression (IHC H–score) using a Spearman rank test.

#### Cluster analysis of gene expression profiles

An unsupervised hierarchical cluster analysis was applied to all samples evaluable for mRNA expression (n = 51), using the preselected list of 1,030 variable genes [15]. A second cluster analysis was performed on the subgroup of patients with a histological diagnosis of adenocarcinoma (n = 47), based on a subset of 870 genes overlapping with the Wilkerson dataset [16]. The identified clusters of samples and gene expression levels were presented in a heatmap along with the profiles of additional relevant binary covariates: PFS (high or low based on the study median PFS of 5.5 months); OS (high or low based on the study median OS of 9.6 months); nuclear and cytoplasmic TS protein expression (high or low determined by optimal H-score cutpoint of 70 and 100, respectively); TS mRNA expression (high or low determined by optimal cutpoint of delta Cq of −1.3) [7]; ECOG performance status (1 vs. 0); age (≥65 years or <65 years); and sex (male or female). Further, Kaplan-Meier estimates of PFS and OS were provided for each cluster, including median PFS and OS and the respective 95% confidence intervals (CIs). Pairwise comparisons between clusters based on HRs or p-values were not calculated because the sample size in each cluster was too small.

## Results

### Patient population and qualified samples

A total of 70 patients were enrolled and started pemetrexed/cisplatin treatment (median age 65.1 years; male/female 54.3/45.7%; Stage IIIb/IV 10.0/90.0%; ECOG performance status 0/1 34.3/65.7%; adenocarcinoma 87.1%). FFPE tumor tissue samples were available from 68 patients; 60 patients with valid TS IHC H-scores had been included in the primary analysis of the study and were incorporated in these analyses as well. Total RNA was extracted from 64 FFPE samples; 59 RNA samples had sufficient quality to be used for microarray profiling. Raw profiling data showed strong signal strengths. Of the 59 microarray samples assessed, 53 (89.8%) had at least 30% of the probe sets present. Eight samples failed quality assessment; valid LC-DSA gene expression profiles were thus available for 51 of the 70 patients (72.9%) enrolled (Figure S2).

### Association of Individual Gene Expression Markers with PFS and OS

Among the 1,030 differentially expressed genes analyzed, 9 were significantly associated with both PFS and OS (Cox regression analysis, unadjusted p<0.01; Figure S3), for both continuous analyses (Table 1) and dichotomous analyses (Table 2). For 8 of these 9 genes, higher expression was significantly associated with longer PFS and OS, and was negatively correlated with nuclear TS-IHC expression (unadjusted p<0.01 for 5 of these 8 genes, Table 1). Of the 9 genes, 5 are known to be potentially linked to adenocarcinoma histology: NK2 homeobox 1 (NKX2-1), aquaporin 4 (AQP4), Chromosome 16 open reading frame 89 (C16orf89), napsin A (NAPSA), and surfactant protein B (SFTPB). Known functions associated with these genes are listed in Table 3. For none of the 9 genes was a relationship to folate metabolism found in the literature.

**Table 1:** Cox regression analyses of association between gene expression and PFS and OS (HR per 1-unit increase), and correlation with nuclear TS expression for 9 genes significantly associated with both PFS and OS (N = 51 patients with nsNSCLC).

	Association with PFS			Association with OS		Spearman corr. with nuclear TS protein expression (IHC)		
	HR	p-value	FDR	HR	p-value	FDR	R	p-value
Chromosome 16 open reading frame 89 (C16orf89)	0.66	<0.001	0.022	0.65	0.001	0.126	−0.42	0.003*
Napsin A	0.79	<0.001	0.022	0.83	0.001	0.126	−0.41	0.004*
Surfactant protein B	0.82	<0.001	0.022	0.86	<0.001	0.126	−0.37	0.009*
Aquaporin 4	0.76	<0.001	0.024	0.77	0.002	0.126	−0.40	0.005*
TRAF2- and Nck-interacting kinase	0.52	<0.001	0.024	0.57	0.006	0.189	−0.36	0.011
Lysophosphatidyl-cholineacyl-transferase 1	0.69	<0.001	0.032	0.74	0.005	0.189	−0.30	0.038
IL-1 receptor, type II	1.42	<0.001	0.032	1.45	0.003	0.171	+0.29	0.047
NKX2-1 (TTF-1)	0.83	0.001	0.036	0.85	0.006	0.189	−0.24	0.100
ABO glycosyl-transferase	0.66	0.004	0.058	0.57	0.003	0.176	−0.40	0.005*

Derived from mRNA profiling data using the Almac Lung Cancer-DSA microarray (1,030 selected genes) [11]. FDR was calculated across 1,030 genes for each endpoint.

*unadjusted p<0.01 for Spearman correlation between TS nucleus expression assessed by IHC.

Abbreviations: FDR, False Discovery Rate; HR, hazard ratio; IHC, immunohistochemistry; IL, interleukin; N, number of patients; NKX2-1, NK2 homeobox 1; nsNSCLC, non-squamous non-small cell lung cancer; OS, overall survival; PFS, progression-free survival; R, spearman correlation coefficient; TS, thymidylate-synthase; TTF-1, transcription factor 1.

**Table 2:** Cox regression analyses of association between gene expression (high versus low expression; cutpoint: median) and PFS and OS (N = 51 patients with nsNSCLC).

	Association with PFS			Association with OS		
	HR	p-value	FDR	HR	p-value	FDR
Chromosome 16 open reading frame 89 (C16orf89)	0.30	<0.001	0.025	0.34	0.002	0.307
Napsin A	0.37	0.002	0.059	0.38	<0.001	0.307
Surfactant protein B	0.24	<0.001	0.023	0.37	0.002	0.307
Aquaporin 4	0.32	<0.001	0.034	0.30	0.008	0.339
TRAF2- and Nck-interacting kinase	0.28	<0.001	0.023	0.25	0.003	0.339
Lysophosphatidyl-cholineacyl-transferase 1	0.31	<0.001	0.045	0.36	0.001	0.307
IL-1 receptor, type II	2.54	0.004	0.092	2.54	0.005	0.339
NKX2-1 (TTF-1)	0.37	0.001	0.052	0.32	0.004	0.339
ABO glycosyl-transferase	0.38	0.002	0.068	0.33	<0.001	0.168

Derived from mRNA profiling data using the Almac Lung Cancer-DSA microarray (1,030 selected genes) [11]. FDR was calculated across 1,030 genes for each endpoint and biomarker type.

Abbreviations: FDR, False Discovery Rate; HR, hazard ratio; IL, interleukin; mRNA, messenger ribonucleic acid; N, number of patients; NKX2-1, NK2 homeobox 1; nsNSCLC, non-squamous non-small cell lung cancer; OS, overall survival; PFS, progression-free survival; TTF-1, transcription factor 1.

**Table 3:** Overview of the 9 genes showing consistent association with both PFS and OS (n = 51 patients with nsNSCLC).

Gene	Known relationshipto ADC	Function (literature information)
Chromosome 16 openreading frame 89 (C16orf89)	Yes	Protein predominantly expressed in human thyroid tissue with specificity intermediate between TTFs and proteins involved in thyroid hormone synthesis [17]
		Shows the same expression pattern as NKX2-1 [17]
Napsin A (NAPSA)	Yes	Gene for an aspartic peptidase, is considered as potential new biomarker for primary lung adenocarcinoma (more specific and sensitive than NKX2-1) [18]; clinical and pathological characteristics of napsin A-positive lung adenocarcinomas are similar to and overlap with those of NKX2-1 positive adenocarcinomas [19]
Surfactant protein B (SFTPB)	Yes	Hydrophobic protein, important for surfactant function and homeostasis [20]
		SP-B mRNA detected in 53% of pulmonary adenocarcinomas with acinar, papillary, bronchioloalveolar, and solid growth patterns. SCLC, LSCLC, and non-pulmonary adenocarcinomas did not contain SP-B mRNA [20]
		NKX2-1 is crucial for branching morphogenesis during normal lung development and transactivates the expression of the surfactant proteins such as SP-A, -B, and -C [21]
Aquaporin 4 (AQP4)	Yes	Potentially involved in lung cancer tumor cell extravasation and spread [22]
		AQP4 expressed constitutively in 40% of all NSCLC carcinomas [23]
		Higher transcript/protein levels of AQP4 in well-differentiated lung adenocarcinomas [23] suggesting association with favorable prognosis
		Microarray summary data published which correlates with the SFTPB, NAPSA, and NKX2-1 genes [23]
TRAF2- and Nck-interactingkinase (TNIK)	No	Member of the germinal center kinase family; involved in cytoskeleton organization [24]
		Emerging evidence indicating that TNIK is essential in activation of WNT pathway [25]
Lysophosphatidylcholine acyltransferase 1 (LPCAT1)	No	Expressed ubiquitously in almost all types of tissues, but is particularly high in surfactant-producing lung type II epithelial cells [26]
Interleukin 1 receptor,type II (IL1R2)	No	Unknown association with NSCLC
		Acts as a negative and thus antiinflammatory regulator for IL-1, inhibits the activity of its ligands, no signaling [27]
		Binds IL-1alpha, IL-1beta [28]
NK2 homeobox 1 (NKX2-1)	Yes	Gene for tissue-specific TTF-1, expressed mainly in the epithelial cells of the lungs and thyroid [29]
		Implicated as contributing to lung cancer development [30]
		Commonly used as marker for the diagnosis of primary and metastatic lung adenocarcinoma [29], [31]
		Potential prognostic marker to explain the histotype-associated pemetrexed activity [32]
ABO glycosyl-transferase	No	Unknown association with NSCLC
		Gene for ABO glycosyltransferase, catalyzes the transfer of carbohydrates to the H antigen, forming the antigenic structure of the ABO blood groups [33]

Abbreviations: ADC, adenocarcinoma; IL, interleukin, LSCLC, large-cell lung cancer; mRNA, messenger ribonucleic acid; nsNSCLC, non-squamous non-small cell lung cancer; SCLC, small-cell lung cancer; SP-A/B/C, surfactant protein A/B/C; TTF-1, transcription factor 1.

### Cluster analysis

**Figure 1:**
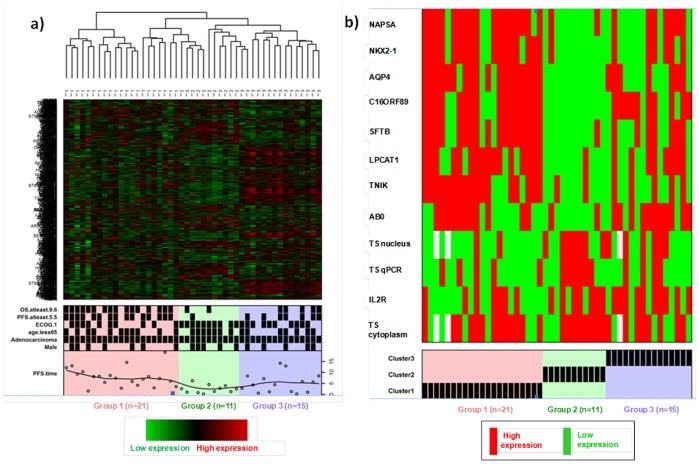
Cluster analysis: Heatmap profiles of adenocarcinoma patients with microarray mRNA data (n = 47). 1a: Columns of the heatmap represent the samples with annotated cluster membership. Rows of the heatmap represent the 870 genes on the Lung Cancer-DSA microarray in common with the set of genes used by Wilkerson et al. [2012]. Additional binary profiles are plotted for the following covariates: male, adenocarcinoma yes, ECOG performance status = 1, PFS ≥5.5 months (median), OS ≥9.6 months (median), PFS time is plotted as an additional profile with added loess-smoothed curve. 1b: Binary profiles plotted for TS nucleus IHC H-score high (≥70), TS cytoplasm IHC H-score high (≥100), TS qPCR high (≥-1.3), and high expression (≥ median cutpoint) of each of the 9 genes significantly associated with both PFS and OS in the overall sample. Abbreviations: ECOG, Eastern Cooperative Oncology Group; n, number of patients; nsNSCLC, nonsquamous non-small cell lung cancer; OS, overall survival; PFS, progression-free survival; qPCR, real-time quantitative polymerase chain reaction; TS, thymidylate synthase.

Cluster analysis of all 51 samples based on 1,030 genes revealed no clear trend for an association between gene expression profile and PFS or OS (data not shown). The cluster analysis of 47 adenocarcinoma samples, based on a subset of 870 genes overlapping with the adenocarcinoma dataset previously described by Wilkerson and coworkers [16], identified 3 distinct groups of 21, 11, and 15 patients respectively. These 3 groups were distinct in terms of PFS, OS, nuclear TS IHC expression, and TS qPCR gene expression (Figure 1, Table 4). Median (95%CI) PFS was 8.1 (6.9, NE), 2.4 (1.2, NE), and 4.4 (1.2, NE) months in Groups 1, 2, and 3, respectively; median (95%CI) OS was 20.3 (17.5, NE), 4.3 (1.4, NE), and 8.3 (3.9, NE) months, respectively. Group 1 included higher proportions of patients with PFS and OS ≥ the median PFS and OS of the study, and low nuclear TS IHC, when compared to Groups 2 and 3. Regarding clinical staging, 4 patients had Stage IIIb adenocarcinoma, these were all in Group 1. All other patients had Stage IV disease (Table 4).

**Table 4:** Profiles of 3 patient clusters identified by hierarchical cluster analysis of 47 adenocarcinoma NSCLC samples.

	Cluster 1 (n = 21)	Cluster 2 (n = 11)	Cluster 3 (n = 15)
Median PFS (95% CI), months	8.1 (6.9, NE)	2.4 (1.2, NE)	4.4 (1.2, NE)
PFS ≥5.5 months, n (%)	15 (71.4)	1 (9.1)	7 (46.7)
Median OS (95% CI), months	20.3 (17.5, NE)	4.3 (1.4, NE)	8.3 (3.9, NE)
OS ≥9.6 months, n (%)	16 (76.2)	2 (18.2)	5 (33.3)
Low TS nucleus IHC H-score (<70), n (%)	17 (89.5)^a^	4 (36.4)	8 (57.1)^b^
Low TS cytoplasm IHC H-score (<100), n (%)	6 (31.6)^a^	3 (27.3)	6 (42.9)^b^
Low TS qPCR (<−1.3), n (%)	16 (76.2)	5 (45.5)	11 (73.3)
Tumor Stage IIIb	4 (19.1)	0	0
Tumor Stage IV	17 (81.0)	11 (100.0)	11 (100.0)

a based on n = 19 patients with data available.

b based on n14 patients with data available.

Abbreviations: CI, confidence interval; IHC, immunohistochemistry; n, number of patients; NE, not estimable; NKX2-1, NK2 homeobox 1; NSCLC, non-small cell lung cancer; OS, overall survival; PFS, progression-free survival; qPCR, real-time quantitative polymerase chain reaction; TS, thymidylate synthase.

## Discussion

In addition to the primary publication assessing the association between TS expression and PFS [7], this publication provides the first gene expression profiling data from a single-arm study of patients treated with pemetrexed and cisplatin, which was a secondary objective of the study. Careful planning and utilization of available tissue material from routine biopsy material allowed us to perform multiple analyses, and results were consistent for PFS and OS across the analyses (continuous and dichotomous).

The primary results showed a statistically significant association between low nuclear TS protein expression and better clinical outcomes in nsNSCLC patients treated with pemetrexed plus cisplatin [7]. The exploratory gene expression profiling data reported here provide additional insights on key genes potentially linked to low TS expression. Among the 1,030 highly variable lung-cancer related genes selected from the array, 9 were significantly associated with both PFS and OS. For 8 of the 9 genes, higher expression was significantly associated with longer PFS and OS, and was negatively correlated with nuclear TS expression (significant for 5 of these 8 genes). Among the 9 genes, 5 have been associated with adenocarcinoma in the literature (NKX2-1, aquaporin 4, C16orf89, napsin A, and surfactant protein B; Table 3), suggesting a potential prognostic role.

Wilkerson et al. identified and characterized 3 molecular subtypes of lung adenocarcinoma (bronchioid, magnoid, and squamoid) [16], [34]. Substantial molecular and clinical differences were observed among the 3 subtypes with different sets of genes being overexpressed in each subtype. The bronchioid subtype was characterized by overexpression of genes involved in excretion, asthma genes and surfactants and by longer OS [16]. In our study a cluster analysis based on 47 adenocarcinoma samples using a gene set overlapping with the gene set used by Wilkerson et al. also identified 3 different groups of patients. PFS and OS were longest in the cluster with low nuclear TS IHC, consistent with the results based on the individual gene level analysis, and showing a similar pattern of survival data as described by Wilkerson et al [16].

This study utilized the disease-specific Lung Cancer DSA microarray which includes approximately 60,000 different transcripts. The most variable probe sets were identified and summarized to a list of 1,030 genes.

The primary results of this single-arm, exploratory study provide the best evidence so far (based on both IHC and qPCR data) that low TS expression is associated with better clinical outcome for patients with NSCLC. Our single-arm study was not designed for comprehensive biomarker research or detection of validated biomarkers, and the sample size was small with only 51 validated mRNA samples available. The exploratory gene expression profiling data presented here identified 9 additional genes that were significantly associated with PFS and OS. However, a literature search did not indicate a relationship for any of these 9 genes and any folate pathway. Known folate metabolism genes were not identified during the exploratory profiling research reported here since they failed to pass the filtering process applied to identify the most variable probe sets.

For 8 of the 9 additional genes identified here, higher expression was associated with longer PFS and OS and was correlated negatively with TS expression. The analysis of TS expression at the protein and gene level, and the exploratory data presented here all showed consistent results across different assays and clinical outcomes. However, due to the limitations of the study design, the associations between gene expression patterns and clinical outcomes observed cannot be differentiated as prognostic or predictive for response to either pemetrexed or cisplatin therapy.

Despite considerable progress in the development of targeted agents in nsNSCLC, the majority of patients are not suitable to receive molecularly targeted therapy, as they do not express any currently known genetic alteration that would make them eligible for such therapy. For these patients, pemetrexed-based treatment remains an acceptable option. Until a well-validated biomarker is identified, and prospectively tested in randomized clinical trials, histology should remain the standard to select metastatic NSCLC patients eligible for treatment with pemetrexed.

## Supporting Information

Figure S1 **Study design.** CR, complete response; IV, intravenous infusion; N, maximum number of patients planned; NSCLC, non-small cell lung cancer; PR, partial response; SD, stable disease; Vit = vitamin.
(TIF)

Figure S2 **Quality assessment of gene expression profiling samples.** Array data were assessed using the EQC program, which utilizes a distribution based analysis using.rpt files with appropriate thresholds and confidence limits iteratively derived from inspection of resultant distributions. Samples failing this initial EQC assessment underwent rehybridization and re-assessment. For samples passing the initial EQC or re-assessment, the array images were subjected to AIA to identify any major artifacts affecting the data. Finally, the data were subjected to batch analysis and data integrity checks, which were performed using n-way analysis of variance and PCA respectively. Abbreviations: AIA, array image analysis; EQC, Almac Extended QC; RNA, ribonucleic acid; PCA, Principal Components Analysis.
(TIF)

Figure S3 **Number of significant associations (unadjusted p-value <0.01) identified between the RMA expression of individual genes on the Lung Cancer-DSA microarray and PFS and OS (N = 51 patients; Cox regression analyses including RMA gene expression either as continuous or as dichotomous variable).** Dichotomous analysis: The respective median value of the gene is used as cut-point). Both analyses combined: Significant association in both continuous and dichotomous analysis. None of the genes was significantly associated with PFS or OS at the 0.01 level when p-values were adjusted for multiple testing using the FDR procedure. Abbreviations: FDR, False Discovery Rate; N, number of patients; NSCLC, non-small cell lung cancer; OS, overall survival; PFS, progression-free survival; RMA, Robust Multichip Average.
(TIF)

File S1 **Feature Selection.**
(DOCX)
