# Supplementary material for: Association between Gene Expression Profiles and Clinical Outcome of Pemetrexed-Based Treatment in Patients with Advanced Non-Squamous Non-Small Cell Lung Cancer: Exploratory Results from a Phase II Study
Source: PLoS One. 2014 Sep 24;9(9):e107455. doi: 10.1371/journal.pone.0107455 (PMC4175467; doi:10.1371/journal.pone.0107455)
Supplement: File S1 — Feature Selection. (DOCX) [file pone.0107455.s004.docx]

**Feature Selection****:** A combined background and variance filter was applied to the data matrix to identify the most variable genes using an in-house developed feature selection program. Firstly, a background filter was applied to remove genes with expression values too low to be distinguished from the background noise. A high threshold was used to remove a large number of probe sets and ensure these probe sets are highly expressed (threshold: α=0.001).

Background expression filter:

Expression values are defined as follows: *E*=median(log_2_ (*I*)) where:

E = Expression

*I* = Intensity

Expressions values must be above the user defined significance level α.

E > log_2_(*z*_α_ σ*Bg*)

E = Expression

σBg = standard deviation of the background

*z*_α_= Quantile of the standard normal distribution (corresponding to α value).

α = Significance level

Background variance filter:

Expressions values must be above the threshold based on σBg.

Log_2_(var_E_) >2[log_2_(σBg) – *E –* log_2_ (log(2))]

var_E_ = Expression variance

σBg = standard deviation of the background

E = Expression

Secondly, an intensity dependant variance filter was applied to the data matrix to remove probe sets with low variance across all samples (threshold: α =1.28 x 10^-12^).

This method filters variance to be above the expression dependent geometrical mean variance by a value specified by significance parameter α as follows:

$$\left\{\left\langle\log_{2}\left(\mathrm{var}_{E}\right)\right\rangle\right\}_{Linear}=b_{1}+b_{2}E$$

var_E_ = Expression variance

*z*_α_ = Quantile of the standard normal distribution (corresponding to α value).

σ(var_E)_ = standard deviation of the expression variance

*b*1 and *b*2 = coefficients of linear regression of log2(var*E*)

Expressions values must be above the user defined significance level α.

***Results – Feature selection***

Feature selection resulted in 1462 most variable probe sets.
